# Low disease activity of microscopic polyangiitis in patients with anti-myosin light chain 6 antibody that disrupts actin rearrangement necessary for neutrophil extracellular trap formation

**DOI:** 10.1186/s13075-022-02974-9

**Published:** 2022-12-16

**Authors:** Miku Yoshinari, Yuka Nishibata, Sakiko Masuda, Daigo Nakazawa, Utano Tomaru, Yoshihiro Arimura, Koichi Amano, Yukio Yuzawa, Ken-Ei Sada, Tatsuya Atsumi, Hiroaki Dobashi, Hitoshi Hasegawa, Masayoshi Harigai, Seiichi Matsuo, Hirofumi Makino, Akihiro Ishizu

**Affiliations:** 1 Department of Medical Laboratory Science, Faculty of Health Sciences, Hokkaido University, Sapporo, Japan; 2 Department of Rheumatology, Endocrinology and Nephrology, Faculty of Medicine and Graduate School of Medicine, Hokkaido University, Sapporo, Japan; 3 Department of Surgical Pathology, Hokkaido University Hospital, Sapporo, Japan; 4 Department of Nephrology and Rheumatology, Kyorin University School of Medicine, Tokyo, Japan; 5 Kichijoji Asahi Hospital, Tokyo, Japan; 6 Department of Rheumatology and Clinical Immunology, Saitama Medical Center, Saitama Medical University, Saitama, Japan; 7 Department of Nephrology, Fujita Health University School of Medicine, Toyoake, Japan; 8 Department of Nephrology, Rheumatology, Endocrinology and Metabolism, Okayama University Graduate School of Medicine, Dentistry and Pharmaceutical Sciences, Okayama, Japan; 9 Department of Clinical Epidemiology, Kochi Medical School, Kochi University, Nankoku, Japan; 10 Division of Hematology, Rheumatology and Respiratory Medicine, Department of Internal Medicine, Faculty of Medicine, Kagawa University, Kagawa, Japan; 11 Department of Hematology, Clinical Immunology and Infectious Diseases, Ehime University Graduate School of Medicine, Ehime, Japan; 12 Division of Rheumatology, Department of Internal Medicine, Tokyo Women’s Medical University School of Medicine, Tokyo, Japan; 13 Tokai National Higher Education and Research System (THERS), Nagoya, Japan; 14 Okayama University, Okayama, Japan

**Keywords:** MPA, NETs, ANETA, Anti-MYL6 antibody

## Abstract

**Background:**

Neutrophil extracellular traps (NETs) are critically involved in microscopic polyangiitis (MPA) pathogenesis, and some patients with MPA possess anti-NET antibody (ANETA). Anti-myosin light chain 6 (MYL6) antibody is an ANETA that affects NETs. This study aimed to determine the significance of anti-MYL6 antibody in MPA.

**Methods:**

The influence of anti-MYL6 antibody on NET formation and actin rearrangement necessary for NET formation was assessed by fluorescent staining. An enzyme-linked immunosorbent assay was established to detect serum anti-MYL6 antibody, and the prevalence of this antibody in MPA was determined. Furthermore, the disease activity and response to remission-induction therapy of MPA were compared between anti-MYL6 antibody-positive and anti-MYL6 antibody-negative MPA patients.

**Results:**

Anti-MYL6 antibody disrupted G-actin polymerization into F-actin, suppressing phorbol 12-myristate 13-acetate-induced NET formation. Serum anti-MYL6 antibody was detected in 7 of 59 patients with MPA. The Birmingham vasculitis activity score (BVAS) of anti-MYL6 antibody-positive MPA patients was significantly lower than anti-MYL6 antibody-negative MPA patients. Among the nine BVAS evaluation items, the cutaneous, cardiovascular, and nervous system scores of anti-MYL6 antibody-positive MPA patients were significantly lower than anti-MYL6 antibody-negative MPA patients, although other items, including the renal and chest scores, were equivalent between the two groups. The proportion of patients with remission 6 months after initiation of remission-induction therapy in anti-MYL6 antibody-positive MPA patients was significantly higher than in anti-MYL6 antibody-negative MPA patients.

**Conclusions:**

Collective findings suggested that anti-MYL6 antibody disrupted actin rearrangement necessary for NET formation and could reduce the disease activity of MPA.

**Supplementary Information:**

The online version contains supplementary material available at 10.1186/s13075-022-02974-9.

## Background

Microscopic polyangiitis (MPA) is an anti-neutrophil cytoplasmic antibody (ANCA)-associated small-vessel vasculitis typically with myeloperoxidase (MPO)-ANCA in serum [1, 2]. MPO-ANCA and MPO-ANCA-induced neutrophil extracellular traps (NETs) play critical roles in MPA pathogenesis [3, 4].

NETs are extracellular web-like substances that consist of unraveled DNA coating with antimicrobial proteins released from activated neutrophils [5]. Although NETs are essential for innate immunity, an excessive NET formation has adverse aspects, such as cytotoxicity [6], thrombogenicity [7], and autoantigenicity [8]. Therefore, NETs are adequately degraded after accomplishing their roles [9].

Recent studies have demonstrated that some patients with MPA possess antibodies to NETs [anti-NET antibody (ANETA)] [10–12]. Currently, ANETA can be detected only by indirect immunofluorescence tests using NETs as substrates, which bind ANETA more strongly than neutrophils used as substrates. Some ANETA can affect NET generation and degradation [10–13]. More recently, myosin light chain 6 (MYL6) has been identified as an antigen of ANETA affecting NET formation [14].

MYL6 is one of the nonphosphorylatable alkali light chains of myosin that mediates the morphological alteration and movement of cells by interacting with F-actin. F-actin is a cytoskeletal filamentous protein formed by the polymerization of spherical G-actin as a monomer. G-actin polymerization into F-actin is required for NET formation [15]. In contrast, F-actin degradation is essential for NET formation [16]. Although actin dynamics during NET formation has not been fully revealed, it can be assumed that anti-MYL6 antibody disturbs actin dynamics in which F-actin is involved and consequently affects NET formation.

This study first assessed the influence of anti-MYL6 antibody on NET formation and actin rearrangement *in vitro*. Next, an enzyme-linked immunosorbent assay (ELISA) was established to detect serum anti-MYL6 antibody, and the prevalence in patients with MPA was determined. Lastly, the association of anti-MYL6 antibody with the disease activity and response to remission-induction therapy of MPA was examined.

## Materials and methods

### NET induction under the presence of anti-MYL6 antibody

After obtaining written informed consent, peripheral blood (10 mL) was drawn from healthy volunteers. Neutrophils extracted from blood using Polymorphprep (Axis-Shield, Dundee, Scotland) were suspended in RPMI 1640 medium containing 10% fetal bovine serum (1×10^6^/mL), seeded in chambers of four-well chamber slides (400 μL/well), and preincubated for 30 min at 37°C. Cells were exposed to 20 nM phorbol 12-myristate 13-acetate (PMA; Sigma-Aldrich, St. Louis, MO, USA) with 0.5 μg/mL anti-human MYL6 polyclonal antibody (rabbit IgG; Abcepta, San Diego, CA, USA) or rabbit IgG (Abcam, Cambridge, UK) as a control for 4 h at 37°C. After rinsing with phosphate-buffered saline (PBS), the samples were mounted with a mounting solution containing 4′,6-diamidino-2-phenylindole (DAPI; Vector Laboratories, Burlingame, CA, USA). A previous study demonstrated that PMA-induced extracellular DNA included the NET marker citrullinated histone H3 [17].

### Fluorescent staining for actin

NETs were induced in peripheral blood neutrophils by PMA with or without anti-MYL6 antibody as above. Before and 30 min, 1 h, and 3 h after incubation, the samples were fixed with 4% paraformaldehyde for 15 min and permeated with 0.5% Triton X-100 for 5 min at room temperature (RT). Thereafter, the samples were reacted with 1:100 dilution of anti-β-actin monoclonal antibody (mAb; mAbcam 8226, mouse IgG1; Abcam) or equivalent concentrations of isotype control mouse IgG1 (Abcam) at 4°C overnight. After rinsing with PBS, the samples were reacted with 4 μg/mL Alexa Fluor 488-conjugated goat anti-mouse IgG1 antibody (Abcam) and 100 nM Acti-stain 555 phalloidin (Cytoskeleton, Denver, CO, USA) for 1 h at RT in the dark. In cells, actin exists in two different forms: nonpolymerized granular form (G-actin) and polymerized filamentous form (F-actin). Anti-β-actin antibody recognizes G-actin [18], whereas phalloidin binds specifically to F-actin. The samples were finally mounted with the mounting solution containing DAPI. This preliminary assay revealed that G-actin polymerization into F-actin occurred in early during NET formation (0–30 min after PMA stimulation), followed by F-actin degradation (Fig. S1). These findings were consistent with previous reports of Stojkov et al. [15] and Metzler et al. [16].

### Patients and healthy controls (HCs)

This study enrolled 59 patients with MPA in an observational cohort of remission induction therapy in Japanese patients with ANCA-associated vasculitis (AAV) and rapidly progressive glomerulonephritis (RPGN), RemIT-JAV-RPGN cohort [19]. Fifteen patients with granulomatosis with polyangiitis (GPA) and 18 patients with eosinophilic granulomatosis with polyangiitis (EGPA) in the same cohort were included as other AAV controls. Nine volunteers were enrolled as HCs.

### Serum preparation

After acquiring written informed consent, peripheral blood (10 mL) was taken without anticoagulants, and blood was centrifuged at 1900 *g* for 15 min at RT for serum separation. The serum samples were stored at −20°C before use. All patients in the RemIT-JAV-RPGN cohort were newly diagnosed with AAV, and blood was drawn from patients with active disease before treatment. Most patients received remission-induction therapy (glucocorticoid with cyclophosphamide or glucocorticoid alone) and maintenance therapy (tapered glucocorticoid with azathioprine or tapered glucocorticoid alone) based on the discretion of the site clinicians according to the Japanese Ministry of Health, Labour, and Welfare guidelines for AAV treatment [20].

### Clinical parameters

Regarding patients with AAV, the Birmingham vasculitis activity score (BVAS) [21] was accessed before treatment. BVAS comprises nine evaluation items, including general, cutaneous, mucous membranes/eyes, ENT (ear, nose, and throat), chest, cardiovascular, abdominal, renal, and nervous system scores. Antigen specificity of ANCA, blood nitrogen urea (BUN), creatinine (Cr), and C-reactive protein (CRP) was determined at the time of blood sampling. Remission 6 months after treatment initiation was defined as BVAS 0 on two occasions at least 1 month apart according to EULAR recommendations [22].

### Establishment of ELISA plates for anti-MYL6 antibody detection

Recombinant human MYL6 (0.5 μg/mL; Novus Biologicals, Centennial, CO, USA) was applied to a 96-well plate (50 μL/well) overnight at 4°C for immobilization. After washing with ELISA wash buffer (Cell Signaling Technology, Danvers, MA, USA), 1% skim milk was applied (150 μL/well) for 1 h at RT to avoid nonspecific binding of antibodies. As a primary antibody, rabbit anti-human MYL6 polyclonal antibody was applied at 0, 0.1, 0.2, 0.4, 0.8, and 1.6 μg/mL (50 μL/well), and the plate was allowed to settle for 1 h at RT. After washing with the buffer, 1:10,000 dilution of horseradish peroxidase (HRP)-conjugated goat anti-rabbit IgG antibody (Jackson ImmunoResearch, West Grove, PA, USA) was applied as a secondary antibody (50 μL/well), and the plate was allowed to settle for 1 h at RT. After washing with the buffer, 3,3′,5,5′-tetramethylbenzidine solution (SeraCare Life Sciences, Milford, MA, USA) was applied (50 μL/well), and the plate was allowed to settle for 30 min at RT in the dark. Then, 1 M hydrochloric acid was applied (50 μL/well) to stop the reaction. Optical density (OD) was measured at the main wavelength of 450 nm and subwavelength of 620 nm. A reasonably good calibration curve was obtained.

### Quantification of anti-MYL6 antibody in sera of patients with AAV

To detect anti-MYL6 antibody in human serum samples, MYL6-immobilized ELISA plates were employed. Human serum samples (1:100 dilution) and HRP-conjugated rabbit anti-human IgG antibody (1:10,000 dilution; GeneTex, Irvine, CA, USA) were used instead of primary and secondary antibodies, respectively. First, using nine sera of HCs, the cutoff OD value of this ELISA was determined as 0.482 [mean+1.5 standard deviation (SD)]. Next, the serum anti-MYL6 antibody titer of 92 patients with AAV, including 59 with MPA, 15 with GPA, and 18 with EGPA, was determined.

### Comparison of clinical parameters between anti-MYL6 antibody-positive and anti-MYL6 antibody-negative MPA patients

Clinical parameters, including antigen specificity of ANCA, BUN, Cr, CRP, and BVAS (total score and each evaluation item score), were compared between anti-MYL6 antibody-positive and anti-MYL6 antibody-negative MPA patients. The proportion of patients with remission 6 months after initiation of remission-induction therapy was also compared between the two groups.

### Statistics

The Student’s *t*-test or *χ*^2^ test was applied to compare the clinical parameters between anti-MYL6 antibody-positive and anti-MYL6 antibody-negative MPA patients. *P*<0.05 was considered statistically significant.

## Results

### Effects of anti-MYL6 antibody on NET formation and actin rearrangement induced by PMA

**Fig. 1:**
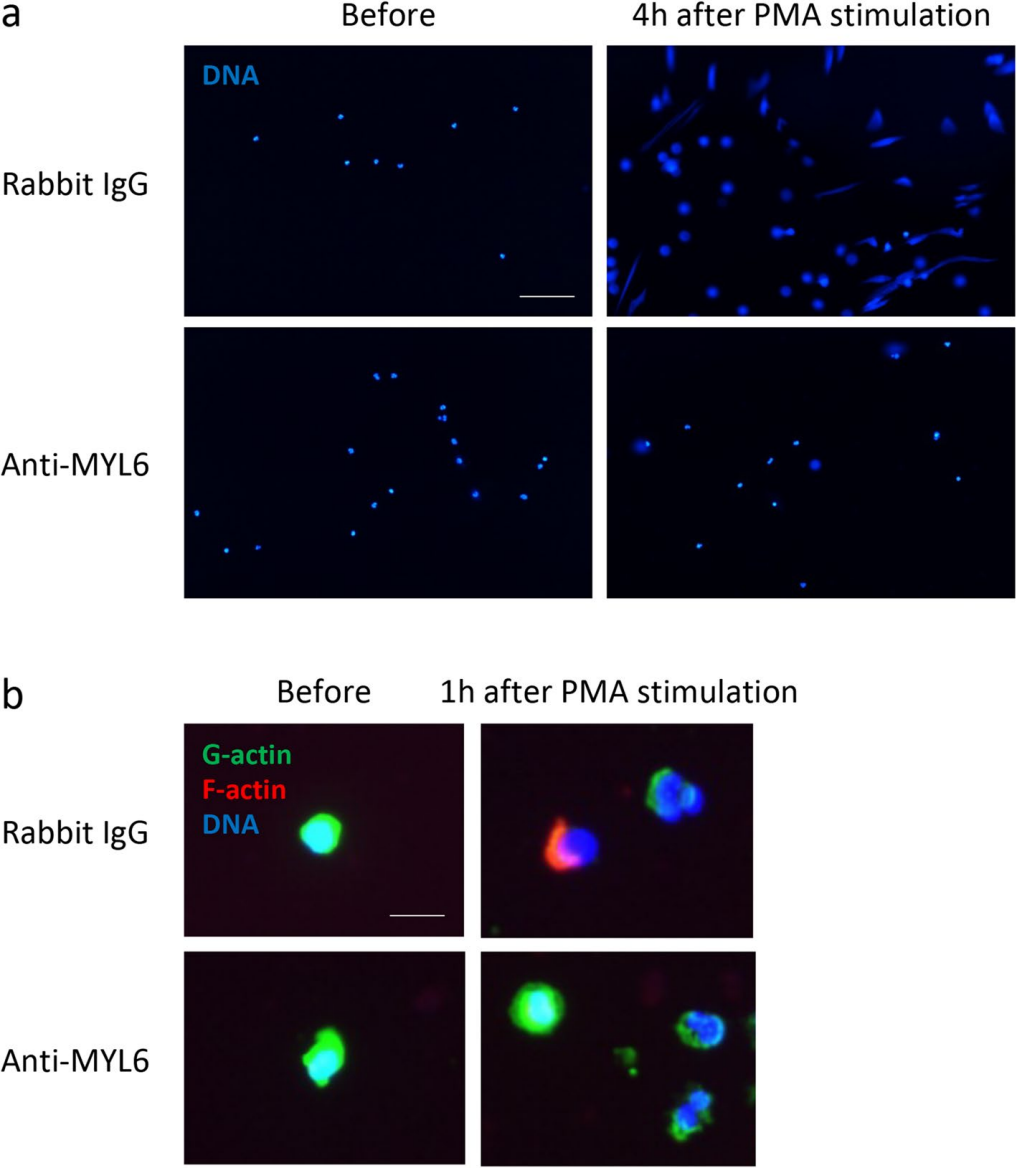
Effects of anti-MYL6 antibody on NET formation and actin rearrangement induced by PMA. **a** Peripheral blood neutrophils from healthy volunteers were stimulated by 20 nM PMA with 0.5 μg/mL anti-human MYL6 polyclonal antibody or rabbit IgG as a control for 4 h at 37°C. After rinsing with PBS, the samples were mounted with a mounting solution containing DAPI. Bar, 100 μm. **b** NETs were induced in peripheral blood neutrophils by PMA with or without anti-MYL6 antibody as above. Before and 1 h after incubation, the samples were fixed with 4% paraformaldehyde for 15 min and permeated with 0.5% Triton X-100 for 5 min at RT. Thereafter, the samples were reacted with 1:100 dilution of anti-β-actin mAb (mouse IgG1) at 4°C overnight. After rinsing with PBS, the samples were next reacted with 4 μg/mL Alexa Fluor 488-conjugated goat anti-mouse IgG1 antibody and 100 nM Acti-stain 555 phalloidin for 1 h at RT in the dark. Cells were finally mounted with the mounting solution containing DAPI. Anti-β-actin antibody recognizes G-actin, whereas phalloidin binds specifically to F-actin. Bar, 10 μm

Extracellular DNA release was observed when neutrophils were stimulated by PMA with the control rabbit IgG, but it was evidently suppressed under the presence of anti-MYL6 antibody (Fig. 1a). Because actin rearrangement occurred early during PMA-induced NET formation (Fig. S1), G- and F-actin distribution in neutrophils stimulated by PMA with or without anti-MYL6 antibody was examined 1 h after incubation. Although F-actin with swollen DNA was observed in a part of neutrophils stimulated by PMA with the control rabbit IgG, it was evidently suppressed, and G-actin remained in neutrophils under the presence of anti-MYL6 antibody (Fig. 1b). These findings suggested that anti-MYL6 antibody could suppress NET formation by disrupting G-actin polymerization into F-actin essential for NET formation.

### Prevalence of anti-MYL6 antibody in MPA

**Fig. 2:**
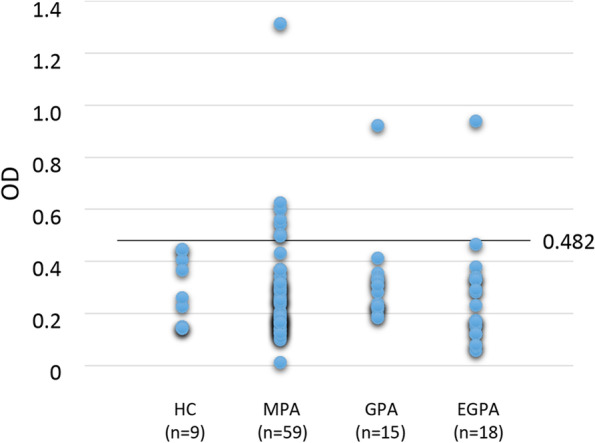
Anti-MYL6 antibody in AAV patients. Anti-MYL6 antibody in sera of 59 patients with MPA was determined by ELISA. As AAV controls, 15 patients with GPA and 18 patients with EGPA were included. The cutoff OD value of this ELISA was determined as 0.482 (mean+1.5 SD) using nine HC samples

The serum titer of anti-MYL6 antibody in 59 patients with MPA, 15 patients with GPA, and 18 patients with EGPA in the RemIT-JAV-RPGN cohort was measured. The comparison of clinical characteristics among three subtypes of AAV is summarized in Table 1. The number of anti-MYL6 antibody-positive patients was 7 in MPA (11.9%), whereas 6.7% (1/15) in GPA and 5.6% (1/18) in EGPA were positive for anti-MYL6 antibody (Fig. 2).

**Table 1:** RemIT-JAV-RPGN cohort patients enrolled in this study

	MPA (*n*=59)	GPA (*n*=15)	EGPA (*n*=18)
Age	69.4±13.0	63.8±20.0	56.8±15.9^a^
Sex (F/M)	28/31	10/5	13/5
BUN (mg/dl)	40.7±24.5	31.7±24.8	13.0±3.5^a^
Cr (mg/dl)	3.19±2.91	2.54±2.46	0.68±0.18^a^
CRP (mg/dl)	6.90±6.89	8.75±6.46	4.85±5.18
BVAS	16.9±4.9	17.0±8.0	18.9±6.9
Immunosuppression (yes/no)	26/33	9/6	6/12
Remission (yes/no)	45/14	13/2	16/2*

^a^*P*<0.01 vs. MPA

*Includes unknown outcome (*n*=1)

### Association of anti-MYL6 antibody and clinical parameters of MPA

**Fig. 3:**
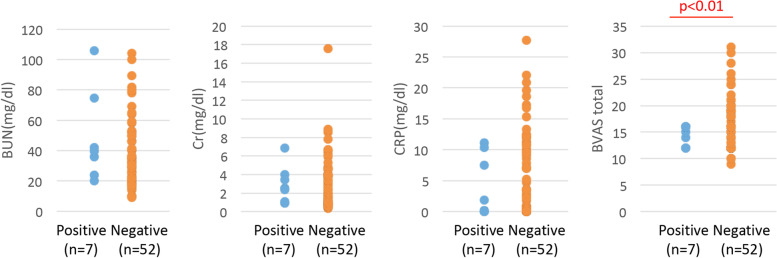
Comparison of clinical parameters between anti-MYL6 antibody-positive and anti-MYL6 antibody-negative MPA patients. Clinical parameters, including BUN, Cr, CRP, and BVAS (total score), were compared between anti-MYL6 antibody-positive and anti-MYL6 antibody-negative MPA patients

**Fig. 4:**
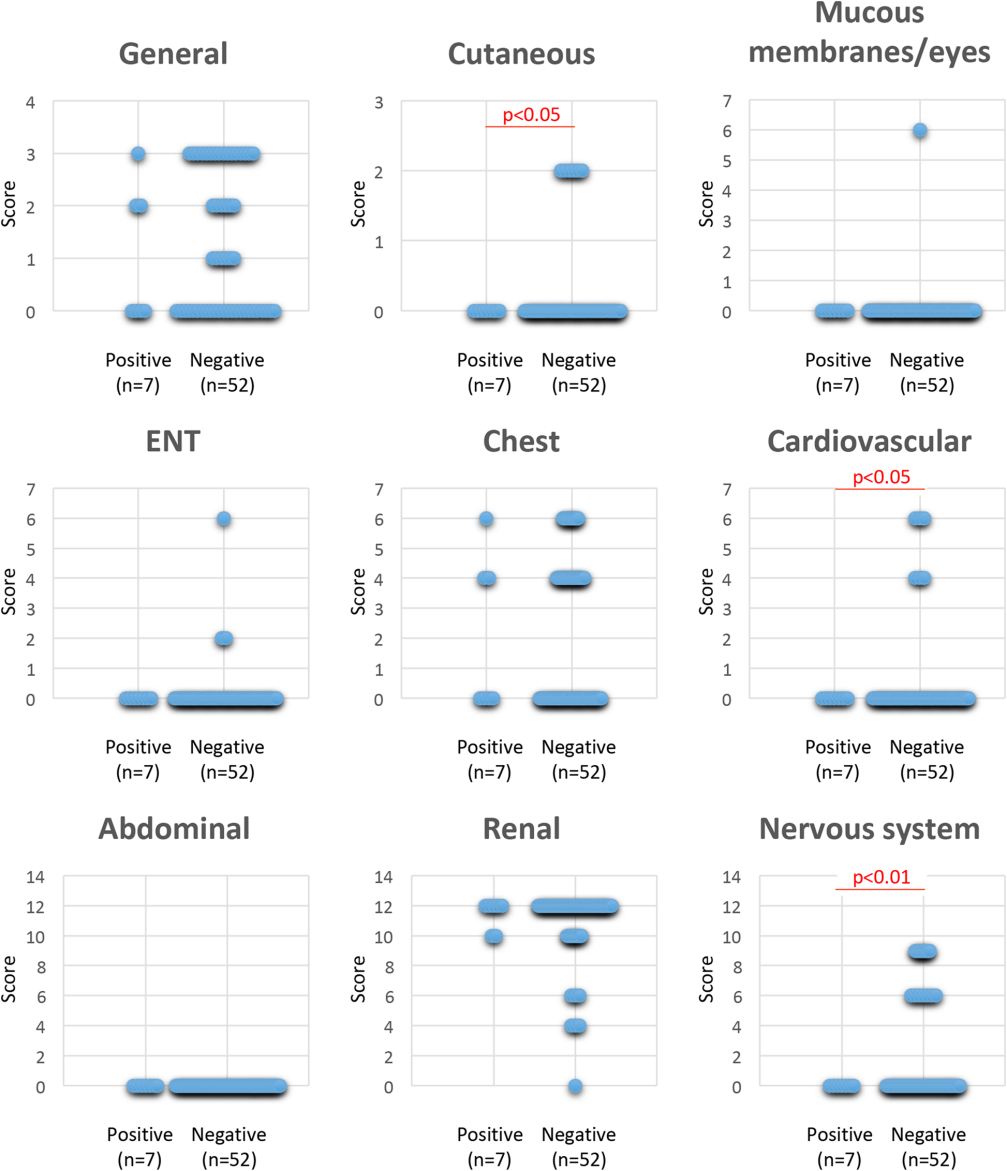
Comparison of nine BVAS evaluation items between anti-MYL6 antibody-positive and anti-MYL6 antibody-negative MPA patients. BVAS evaluation items, including general, cutaneous, mucous membranes/eyes, ENT, chest, cardiovascular, abdominal, renal, and nervous system scores, were compared between anti-MYL6 antibody-positive and anti-MYL6 antibody-negative MPA patients

Clinical parameters, including antigen specificity of ANCA, BUN, Cr, CRP, and BVAS, were compared between anti-MYL6 antibody-positive MPA patients (*n*=7) and anti-MYL6 antibody-negative MPA patients (*n*=52). There was no significant difference in antigen specificity of ANCA between the two groups (Table 2). Among parameters other than ANCA, the BVAS of anti-MYL6 antibody-positive MPA patients was significantly lower than anti-MYL6 antibody-negative MPA patients (14.4±1.8 vs. 17.2±5.1; *p*=0.009; Fig. 3). Concerning the nine evaluation items of BVAS, the cutaneous, cardiovascular, and nervous system scores of anti-MYL6 antibody-positive MPA patients were significantly lower than anti-MYL6 antibody-negative MPA patients (*p*=0.0128, *p*=0.0148, and *p*<0.001, respectively), although other items, including the renal and chest scores, were equivalent between the two groups (Fig. 4). Moreover, the proportion of patients with remission 6 months after initiation of remission-induction therapy in anti-MYL6 antibody-positive MPA patients was significantly higher than in anti-MYL6 antibody-negative MPA patients (*p*=0.014; Table 3).

**Table 2:** Antigen specificity of ANCA between anti-MYL6 antibody-positive and anti-MYL6 antibody-negative MPA patients

MPA (*n*=59)		ANCA specificity		Total
		MPO	PR3	
Anti-MYL6	Positive	7	1^a^	7
	Negative	51	3^b^	52

*PR3* proteinase 3

^a^Includes one double-positive case

^b^Includes two double-positive cases

**Table 3:** Comparison of remission rate 6 months after treatment between anti-MYL6 antibody-positive and anti-MYL6 antibody-negative MPA patients

MPA (*n*=59)		Response to remission-induction therapy		Total
		Remission	No remission	
Anti-MYL6	Positive	7*	0	7
	Negative	38	14	52

*P<0.05

## Discussion

Some MPA patients possessed ANETA [10–12]. A kind of ANETA induced NETs and was thought to be implicated in the relapse of MPA [11]. Others have a NET degradation inhibitory activity, potentially suspected of promoting NET-mediated pathology of MPA [10, 12]. ANETA with NET degradation inhibitory activity has also been reported in antiphospholipid syndrome [13]. In this study, a unique property of ANETA that recognizes MYL6 has been demonstrated.

When neutrophils were stimulated by PMA with anti-MYL6 antibody, NET formation was obviously suppressed. Meanwhile, G-actin did not polymerize into F-actin and remained in neutrophils. Based on these findings, NET induction was suppressed by anti-MYL6 antibody via the disruption of actin rearrangement essential for NET formation [15], and myosin might be required for G-actin polymerization. Further studies need to determine why G-actin polymerization into F-actin is disrupted when the interaction between myosin and F-actin has interfered with anti-MYL6 antibody.

The adverse actions of NETs, including cytotoxicity [6], thrombogenicity [7], and autoantigenicity [8], are critically involved in AAV pathogenesis [4]. Therefore, whether anti-MYL6 antibody with NET formation inhibitory potential could reduce the disease activity of MPA was investigated. This study indicated that the BVAS, especially the cutaneous, cardiovascular, and nervous system evaluation items, of anti-MYL6 antibody-positive MPA patients was lower than anti-MYL6 antibody-negative MPA patients and that the proportion of patients with remission 6 months after initiation of remission-induction therapy in anti-MYL6 antibody-positive MPA patients was significantly higher than in anti-MYL6 antibody-negative MPA patients. These findings were consistent with reports of the involvement of NETs in cutaneous and nervous system lesions in AAV [23, 24] and cardiovascular diseases [25].

Although it is elusive whether anti-MYL6 antibody is exactly associated with good prognosis because MPA patients in the RemIT-JAV-RPGN cohort received diverse treatment, the association of NET formation inhibitory potential of anti-MYL6 antibody and the low activity scores of cutaneous, cardiovascular, and nervous system lesions in anti-MYL6 antibody-positive MPA patients before treatment is suggestive of an involvement of the myosin and F-actin interaction in NET formation leading to the development of these lesions. This may be a clue to understanding the pathology and discovering a novel therapeutic target of MPA.

Collective findings suggested that anti-MYL6 antibody could be a disease-protective autoantibody. Another recent study has demonstrated that anti-serpin B13 autoantibody possesses the potential to prevent type 1 diabetes [26]. Disease-protective autoantibodies will be worthy of attention in upcoming studies.

The limitation of this study includes the small sample number of MPA patients and disease controls. Although the prevalence of anti-MYL6 antibody positivity seemed to be lower in GPA (6.7%) and EGPA (5.6%) than in MPA (11.9%), there was no statistical power. Further studies need to determine whether the disease activity of GPA and EGPA in patients with anti-MYL6 antibody is lower than in anti-MYL6 antibody-negative GPA/EGPA patients. To the authors’ knowledge, anti-MYL6 antibody has not yet been described in the literature. To determine that this autoantibody is specific to MPA or AAV, its production in patients with vasculitis other than AAV and other autoimmune diseases is required as well as in more healthy subjects. In addition, longer observation is required to assess the influence of anti-MYL6 antibody on patient mortality.

## Conclusions

Although further studies are needed, this study considers that anti-MYL6 antibody can disrupt actin rearrangement necessary for NET formation and possibly reduce the disease activity of MPA.

## Supplementary Information

**Additional file 1: Figure S1.** Chronological G- and F-actin distribution in neutrophils stimulated by PMA. NETs were induced in peripheral blood neutrophils by 20 nM PMA. Before and 30 min, 1 h, and 3 h after incubation, the samples were fixed with 4% paraformaldehyde for 15 min and permeated with 0.5% Triton X-100 for 5 min at RT. Thereafter, the samples were reacted with 1:100 dilution of anti-β-actin mAb (mouse IgG1) at 4°C overnight. After rinsing with PBS, the samples were next reacted with 4 μg/mL Alexa Fluor 488-conjugated goat anti-mouse IgG1 antibody and 100 nM Acti-stain 555 phalloidin for 1 h at RT in the dark. The samples were finally mounted with the mounting solution containing DAPI. Anti-β-actin antibody recognizes G-actin, whereas phalloidin binds specifically to F-actin. Bar, 50 μm.

## Data Availability

The data used and/or analyzed during the current study are available from the corresponding author on reasonable request.

## Abbreviations

AAV: ANCA-associated vasculitis
ANCA: Anti-neutrophil cytoplasmic antibody
ANETA: Anti-NET antibody
BUN: Blood nitrogen urea
BVAS: Birmingham vasculitis activity score
Cr: Creatinine
CRP: C-reactive protein
DAPI: 4′,6-Diamidino-2-phenylindole
EGPA: Eosinophilic granulomatosis with polyangiitis
ELISA: Enzyme-linked immunosorbent assay
GPA: Granulomatosis with polyangiitis
HC: Healthy control
HRP: Horseradish peroxidase
mAb: Monoclonal antibody
MPA: Microscopic polyangiitis
MPO: Myeloperoxidase
MYL6: Myosin light chain 6
NETs: Neutrophil extracellular traps
OD: Optical density
PBS: Phosphate-buffered saline
PMA: Phorbol 12-myristate 13-acetate
RT: Room temperature
SD: Standard deviation
