# Supplementary material for: Low disease activity of microscopic polyangiitis in patients with anti-myosin light chain 6 antibody that disrupts actin rearrangement necessary for neutrophil extracellular trap formation
Source: Arthritis Res Ther. 2022 Dec 16;24:274. doi: 10.1186/s13075-022-02974-9 (PMC9756472; doi:10.1186/s13075-022-02974-9)
Supplement: Supplementary file 1 — Additional file 1: Figure S1. Chronological G- and F-actin distribution in neutrophils stimulated by PMA. NETs were induced in peripheral blood neutrophils by 20 nM PMA. Before and 30 min, 1 h, and 3 h after incubation, the samples were fixed with 4% paraformaldehyde for 15 min and permeated with 0.5% Triton X-100 for 5 min at RT. Thereafter, the samples were reacted with 1:100 dilution of anti-β-actin mAb (mouse IgG1) at 4°C overnight. After rinsing with PBS, the samples were next reacted with 4 μg/mL Alexa Fluor 488-conjugated goat anti-mouse IgG1 antibody and 100 nM Acti-stain 555 phalloidin for 1 h at RT in the dark. The samples were finally mounted with the mounting solution containing DAPI. Anti-β-actin antibody recognizes G-actin, whereas phalloidin binds specifically to F-actin. Bar, 50 μm. [file 13075_2022_2974_MOESM1_ESM.pdf]

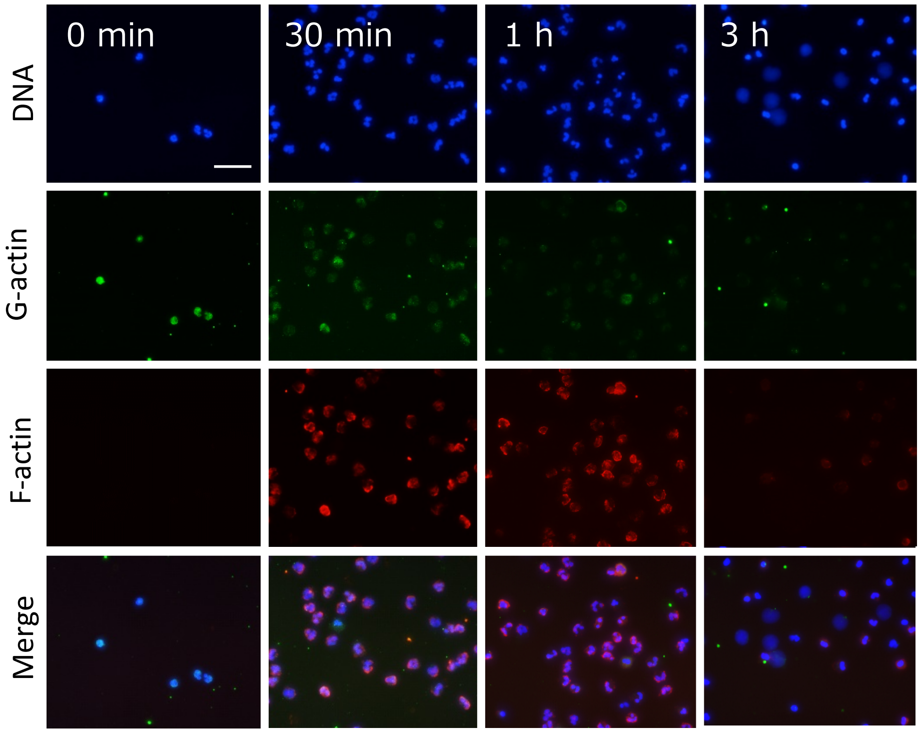

Figure S1. Chronological G- and F-actin distribution in neutrophils stimulated by PMA
NETs were induced in peripheral blood neutrophils by 20 nM PMA. Before and 30 min, 1 h, and 3 h after incubation, the samples were fixed with 4% paraformaldehyde for 15 min and permeated with 0.5% Triton X-100 for 5 min at RT. Thereafter, the samples were reacted with 1:100 dilution of anti-β-actin mAb (mouse IgG1) at 4°C overnight. After rinsing with PBS, the samples were next reacted with 4 μg/mL Alexa Fluor 488-conjugated goat anti-mouse IgG1 antibody and 100 nM Acti-stain 555 phalloidin for 1 h at RT in the dark. The samples were finally mounted with the mounting solution containing DAPI. Anti-β-actin antibody recognizes G-actin, whereas phalloidin binds specifically to F-actin. Bar, 50 μm.
